# Antibody drug conjugates and bystander killing: is antigen-dependent internalisation required?

**DOI:** 10.1038/bjc.2017.367

**Published:** 2017-10-24

**Authors:** Alexander H Staudacher, Michael P Brown

**Affiliations:** 1 Translational Oncology Laboratory, Centre for Cancer Biology, SA Pathology and University of South Australia, Adelaide, SA 5000, Australia; 2 School of Medicine, University of Adelaide, Adelaide, SA 5000, Australia; 3 Cancer Clinical Trials Unit, Royal Adelaide Hospital, Adelaide, SA 5000, Australia

**Keywords:** antibody drug conjugate, bystander killing, non-internalising, cathepsin B

## Abstract

Antibody drug conjugates (ADCs) employ the exquisite specificity of tumour-specific monoclonal antibodies (mAb) for the targeted delivery of highly potent cytotoxic drugs to the tumour site. The chemistry of the linker, which connects the drug to the mAb, determines how and when the drug is released from the mAb. This, as well as the chemistry of the drug, can dictate whether the drug can diffuse into surrounding cells, resulting in ‘bystander killing’. Initially, any bystander killing mechanism of action of an ADC was understood to involve an essential sequence of steps beginning with surface antigen targeting, internalisation, intracellular linker cleavage, drug release, and diffusion of drug away from the targeted cell. However, recent studies indicate that, depending on the linker and drug combination, this mechanism may not be essential and ADCs can be cleaved extracellularly or via other mechanisms. In this minireview, we will examine the role of bystander killing by ADCs and explore the emerging evidence of how this can occur independently of internalisation.

Ideally, anti-cancer treatments should specifically target and kill tumour cells while leaving normal, healthy tissues relatively unscathed. The therapeutic ratio of the maximum tolerated to the minimally efficacious dose of many of the drugs used in conventional cytotoxic chemotherapy is relatively low, resulting in unwanted side-effects. Nevertheless, these drugs are relatively effective against cancer cells because normal cells have more efficient mechanisms for effluxing cytotoxins, repairing DNA, and clearing dead and damaged cells. Together, these mechanisms limit toxicity even among the rapidly dividing cells of the bone marrow and gastrointestinal epithelium before the next cycle of chemotherapy is given. Immunotherapy has now joined cytotoxic chemotherapy as an effective systemic treatment for cancer, and antibody-based therapy has contributed most significantly to this burgeoning area. Monoclonal antibodies (mAbs) may directly stall or kill tumour cells by binding tumour cell-specific antigens, or indirectly kill tumour cells by inhibiting the immune checkpoint molecules that restrain tumouricidal lymphocytes. Building on these advances, antibody drug conjugate (ADC) therapy has emerged with worldwide marketing approvals for the indications of breast cancer and lymphoma.

An ADC is divided into three distinct parts – the tumour-targeting mAb, the linker, and the cyotoxic drug, also known as the payload or warhead (Figure 1). Modern ADC technology is successful because the exquisite specificity of mAbs is stably coupled with the extreme potency of new cytotoxins. Therapeutic mAbs are commonly directed at antigens on the surface of tumour cells themselves or the cells or other components of the supporting tumour stroma. Ideally, these antigens are expressed at minimal levels on normal healthy tissues but are highly expressed by tumour cells or within the tumour microenvironment. The payload drugs used with ADCs are orders of magnitude more potent than conventional cytotoxic drugs, such as chemotherapeutic drugs, and as such are too potent to use safely as free drugs for cancer therapy. Moreover, advances in drug/linker chemistries, which enhance plasma stability and control cytotoxin release at the tumour site, have critically enabled modern ADC technology. Consequently, ADCs are expected to have a higher therapeutic ratio than conventional systemic chemotherapy.

Two ADCs are currently FDA-approved for cancer treatment; ado-trastuzumab emtansine (KADCYLA, Roche/Genentech, San Francisco, CA, USA) for the treatment of patients with human epidermal growth receptor 2 (HER2)-expressing, metastatic breast cancer (Verma *et al*, 2012) and brentuximab vedotin (ADCETRIS, Seattle Genetics, Bothell, WA, USA) for the treatment of relapsed or refractory CD30-expressing Hodgkin lymphoma (HL) (Younes *et al*, 2012) and anaplastic large cell lymphoma (ALCL) (Fanale *et al*, 2012; Pro *et al*, 2012). A large number of early phase clinical trials examining the therapeutic effectiveness of a range of different ADCs are currently underway.

Although several recent reviews on ADCs have been published covering the evolution of ADC technology through different mAbs, linker technologies and drug payloads (such as Diamantis and Banerji, 2016; Polakis, 2016; Lambert and Morris, 2017; Beck *et al*, 2017), information is lacking about the ‘bystander killing’ that can be mediated by ADCs with particular linker and/or payload combinations and whether internalisation of the ADC is an essential part of its mechanism of action. In this minireview, we will focus primarily on bystander killing by ADCs and the mechanism(s) involved. We will illustrate the potential of ADC-mediated bystander killing through the two contrasting clinical examples of ado-trastuzumab emtansine and brentuximab vedotin, and discuss whether direct antigen-mediated internalisation of the ADC is required for bystander killing.

## Bystander killing

Bystander killing occurs when the drug from an ADC is released either from the target cell following internalisation and degradation of the ADC or release of the drug within the extracellular space. In both cases, the drug is then taken up by and kills surrounding or bystander cells, which themselves may or may not express the ADC target antigen. How much an ADC mediates bystander killing depends largely on factors such as the extent of ADC internalisation after binding to the target antigen, the presence of a non-cleavable or cleavable linker, and the hydrophobicity of the attached cytotoxic warhead.

To exert their cytotoxic effects, ADCs such as ado-trastuzumab emtansine, which have a non-cleavable (commonly a thioether) linker, must be internalised by the target cell for the antibody, rather than the linker, to be degraded before release of the drug. This form of ADC does not produce efficient bystander killing because, typically, it contains the potent tubulin-binding maytansine derivative, DM1, and the cleaved drug product cannot enter surrounding cells because its positive charge prevents it from penetrating the cell membrane (Erickson *et al*, 2006). Therefore, non-cleavable ADCs are primarily effective against the target antigen-expressing cell after internalisation and are best suited to treat cancers that have high and homogenous expression of the target antigen (Kovtun *et al*, 2006).

Alternatively, ADCs may contain a cleavable linker, which can be cleaved at a defined pH range or by specific proteases to release the free drug. Although the resulting free drug can directly kill the target cell, it can diffuse out of the target cell to cause bystander killing depending on the drug type and its physicochemical properties (Kovtun *et al*, 2006; Li *et al*, 2016; Singh *et al*, 2016). In spite of a consensus that ADCs are only effective if internalised via a cell-surface antigen, evidence is emerging to indicate that ADCs with particular linker/drug combinations can exert cell killing through other mechanism(s).

### Current FDA-approved ADCs and bystander killing

The FDA-approved ADC, ado-trastuzumab emtansine, comprises the humanised mAb trastuzumab, which targets HER2, an antigen overexpressed in ≈25–30% of breast cancers, with treatment with trastuzumab alone, resulting in partial and complete responses in metastatic breast cancer patients (Cobleigh *et al*, 1999). The drug-conjugated version of trastuzumab, ado-trastuzumab emtansine, consists of trastuzumab bound by a non-cleavable thioether linker to DM1. Once internalised by target cells, the ADC is transported to the endosome where it is degraded, releasing an active but cell-impermeant drug, which kills the targeted cell. The cleaved drug product becomes trapped inside the target cell because it includes a positively charged lysine moiety, which cannot effectively penetrate the cell membrane and cannot therefore elicit bystander killing (Kovtun *et al*, 2006). This ADC provided significant improvement in progression-free survival when compared with trastuzumab in combination with docetaxel, with fewer adverse incidents reported (Hurvitz *et al*, 2013). Furthermore, patients with higher than median tumour expression of HER2 mRNA responded better to ado-trastuzumab emtansine than those with lower than median HER2 mRNA expression (Perez *et al*, 2014), indicating a relationship between the extent of HER2 expression and responsiveness to treatment.

The other FDA-approved ADC uses the chimeric mAb brentuximab to target the CD30 antigen, which is expressed at high density on the malignant Hodgkin and Reed-Sternberg (HRS) cell in HL and on the non-HL cells of ALCL. In contrast, normal tissue expression of CD30 is limited to activated B and T cells (Senter and Sievers, 2012), thymocytes during development, uterine cells during pregnancy, and pancreatic exocrine cells (Ansell, 2014). The ADC, brentuximab vedotin, consists of brentuximab conjugated to the microtubule-binding auristatin, monomethyl auristatin E (MMAE), through a protease-labile, dipeptide valine–citrulline (vc) linker. After binding to CD30, the ADC is internalised and transported to lysosomes where the linker is cleaved to release the free drug. The clinical results are high rates of durable responses among patients with a number of CD30^+^ lymphomas despite great variation in lymphoma cell expression of CD30. Among patients with relapsed or refractory HL (Younes *et al*, 2012) and ALCL (Pro *et al*, 2012), the CD30 target antigen is expressed strongly and uniformly on the neoplastic cells. Notwithstanding the strong and uniform CD30 expression on HL and ALCL cells, it was not apparent that a minimal threshold of CD30 was required for tumour response in the pivotal studies of brentuximab vedotin for these lymphomas (Pro *et al*, 2012, 2013; Younes *et al*, 2012; Jacobsen *et al*, 2015). Conversely, in tumours of patients with T-cell lymphomas and diffuse large B-cell lymphoma (DLBCL), CD30 expression is more variable (Bossard *et al*, 2014; Slack *et al*, 2014; Jacobsen *et al*, 2015).

Nevertheless, the CD30^+^ neoplastic HRS cell of HL represents only a small proportion (0.1–10%) of the total lymphoma cell population, which mainly comprises such reactive cell types as T and B lymphocytes, plasma cells, eosinophils, neutrophils, macrophages, and fibroblasts (Brown and Staudacher, 2014). Hence, unlike ado-trastuzumab emtansine, correlations between tumour CD30 expression and tumour responses to brentuximab vedotin have not been found for other lymphomas with variable expression of CD30 (Fromm *et al*, 2012; Bartlett *et al*, 2013; Horwitz *et al*, 2014; Duvic *et al*, 2015; Jacobsen *et al*, 2015). In a phase 2 study of 35 patients with CD30^+^ relapsed or refractory peripheral T-cell lymphoma who received brentuximab vedotin, there was no apparent correlation between response and tumour CD30 expression; 9 of 14 patients (64%) had responses with little (⩽15%) or no CD30 expression by central review of immunohistochemistry (Horwitz *et al*, 2014). In another example, an ALCL patient, whose skin lesion had an estimated 3% occupancy of CD30-binding sites, obtained a complete remission after treatment with brentuximab vedotin (Fromm *et al*, 2012). Again, in a phase 2 study of 48 patients with CD30^+^ cutaneous T-cell lymphoma, brentuximab vedotin produced a high overall tumour response rate of 73% but the responses did not correlate with tumour CD30 expression; even patients with low-level expression (<10%) achieved 50% partial response rate (Duvic *et al*, 2015).

Finally, there was no statistical correlation between CD30 expression in DLBCL and tumour response to brentuximab vedotin in a phase 2 study, which included 49 relapsed or refractory DLBCL patients, although all the responders had quantifiable CD30 expression by computer assisted digital video analysis immunohistochemistry (cIHC). Remarkably, one patient with relapsed DLBCL, who had 0% CD30 expression within the tumour biopsy by central review of visual IHC and 1.4% via cIHC, still achieved a complete response after two cycles of brentuximab vedotin (Jacobsen *et al*, 2015). In a follow-up study of 52 DLBCL patients with absent CD30 expression by visual IHC, 16 patients (31%) had an objective response including six complete responses (12%). Analysis by cIHC showed that 11 of the 16 DLBCL responders had ⩾1% CD30, and this was associated with significantly longer median overall survival. These data suggest that a minimum threshold of CD30 expression is required for anti-tumour activity of brentuximab vedotin in DLBCL (Bartlett *et al*, 2017).

Together, these data suggest that factors other than direct killing of antigen-positive cells by brentuximab vedotin must be involved as even low CD30-expressing disease can be just as responsive to brentuximab vedotin as high CD30-expressing disease. Bystander killing mechanisms may help to explain these observations (Brown and Staudacher, 2014; Masuda *et al*, 2015). Interestingly, new DNA minor groove binding cytotoxic warheads of picomolar potency, which have been incorporated in ADCs have also been recently reported to exhibit bystander killing effects in pre-clinical models (Flynn *et al*, 2016; Miller *et al*, 2016).

### Is ADC internalisation required for bystander killing?

ADCs with non-cleavable thioether linkers have particular advantages: high stability in serum, manifest anti-tumour activity only after internalisation and lysosomal degradation (Erickson *et al*, 2006), and greatest anti-tumour efficacy with high and homogenous tumour expression of target antigen (Kovtun *et al*, 2006). However, these ADCs do not cause bystander killing because only the tumour cells that have internalised the ADC are killed with little or no effect on surrounding, antigen-negative cells (Kovtun *et al*, 2006).

Cleavable linkers include chemically labile (disulphide and pH-dependent) and enzyme-labile (peptide-based) linkers. It is often presumed that these linkers are cleaved only after internalisation by the target cell with subsequent lysosomal degradation releasing free drug. ADCs with these linkers and appropriate drugs can kill not only the antigen-positive target cells but also the surrounding antigen-negative cells. Consequently, these ADCs are useful for treating tumours with heterogeneous antigen expression (Kovtun *et al*, 2006; Okeley *et al*, 2010; Golfier *et al*, 2014). However, accumulating evidence now suggests that ADC internalisation may not be essential to facilitate tumour cell killing by these particular types of ADC.

#### pH-dependent linkers

Acid-labile or pH-dependent linkers are designed to be stable at the relatively neutral pH of blood but are hydrolysed when in an acidic environment (pH<5). Although degradation of the acid-labile linker in the acidic lysosome is the major degradation pathway for drug release, studies have shown that this may also happen extracellularly. For example, targeting the poorly internalising antigen CD20 with an ADC consisting of the acid-labile 4-(4-acetylphenoxy)butanoic acid (AcBut) linker and the DNA minor groove-binding drug calicheamicin was effective in a preclinical lymphoma model, whereas substitution with an acid-stable (amide) linker had no anti-tumour effect when compared with the parental antibody alone (Dijoseph *et al*, 2007). Furthermore, an antibody directed against extracellular rather than cell-surface mucin, which was conjugated via an acid-labile linker (CL2A) to the active metabolite of irinotecan, also showed an effective response in a preclinical pancreatic cancer model (Sharkey *et al*, 2011). These results suggest that ADCs with an acid-labile linker may not require internalisation for therapeutic potency. The tumour microenvironment, which is much more acidic than normal tissues because of enhanced glycoloysis and lactate generation, may be sufficient for extracellular cleavage of the linker.

#### Disulphide linkers

Disulphide linkers use a direct covalent bond between sulphide groups belonging to the drug and linker. Two linkers, *N*-succinimidyl-4-(2-pyridyldithio)pentanoate (SPP) and N-succinimidyl-4-(2-pyridyldithio)butanoate (SPDB), which contain one or two methyl groups adjacent to the disulphide bond, respectively, resulting in increasing steric hindrance, have been used for ADCs. The more sterically hindered SPDB linker showed superior activity compared with the SPP linker (Erickson *et al*, 2010), and in some cases these cleavable linkers result in more potent ADCs than their counterpart non-cleavable linkers (Polson *et al*, 2009; Erickson *et al*, 2010). Although ADCs using these linkers can cause bystander killing, it is believed that internalisation, reduction of the disulphide bond, and further modification to the drug within the target cell are required for modifying the potency of the drug once released from the cell to cause bystander killing (Erickson *et al*, 2006, 2010; Kovtun *et al*, 2006; Golfier *et al*, 2014).

However, there are studies that indicate that internalisation of these cleavable linker-based ADCs may not be essential for their anti-tumour activity. For example, conjugation of the tubulin-binding maytansine (DM1) to antibodies targeting poorly internalising antigens (CD20, CD21, CD72) via the cleavable SPP linker were effective in xenograft models and were more effective than ADCs with the same antibody/drug using the non-cleavable thioether linker succinimidyl-4-(*N*-maleimidomethyl)cyclohexane-1-carboxylate (SMCC) (Polson *et al*, 2009). In another example, the ADC targeting the carcinoembryonic antigen cell adhesion molecule 5 (CEACAM5) used a cleavable disulphide linker, which is not internalised or slowly internalising, and was effective in the treatment of colon and pancreatic xenografts (Govindan *et al*, 2009).

The recent ‘traceless’ technology uses a direct, unhindered disulphide bond between a drug and an antibody or a small immune protein (SIP). The resulting drug conjugates do not require internalisation by the target cell because the drug is released upon reduction or hydrolysis of the disulphide bond within the tumour microenvironment. This technology has used a SIP format of the F8 antibody, which binds the alternatively spliced extracellular domain A of fibronectin, an extracellular tumour angiogenesis marker. In preclinical studies using (SIP)F8 conjugated to the tubulin-binding drug, cemadotin, which is an analogue of dolastatin and chemically related to the aurostatins, some complete tumour regressions were observed at relatively high daily doses of 43 mg kg^−1^ given for 5–7 days (Bernardes *et al*, 2012; Steiner *et al*, 2013). Conversely, substitution with a more potent maytansanoid (DM1) resulted in much more durable responses at lower administered doses (Perrino *et al*, 2014). The mechanism of action of these ADCs depends on a reducing tumour microenvironment to enable cleavage of the disulphide bonds independently of ADC internalisation because the ADC does not become internalised. It is believed that the release of thiols by dead tumour cells may produce a self-amplifying effect, which further increases the degradation of disulphide bonds of the drug conjugate (Bernardes *et al*, 2012).

More recently, anti-tumour activity of a full-length F8 mAb ADC, which was also conjugated via a direct disulphide bond to the maytansinoid DM1, was observed in a teratocarcinoma xenograft model (Gebleux *et al*, 2015). Strikingly, although the F8 mAb ADC showed superior tumour uptake compared to the F8 SIP ADC (up to fourfold higher) and much greater stability in plasma, it was less effective in controlling tumour growth compared with F8 SIP ADC (one out of five *vs* three out of five complete responders) when given at equivalent molar concentrations (Gebleux *et al*, 2015). In this case, the ‘instability’ of the SIP-drug interaction may actually result in quicker drug release in the tumour resulting in higher drug accumulation.

#### Enzyme-labile (peptide-based) linkers

**Figure 2:**
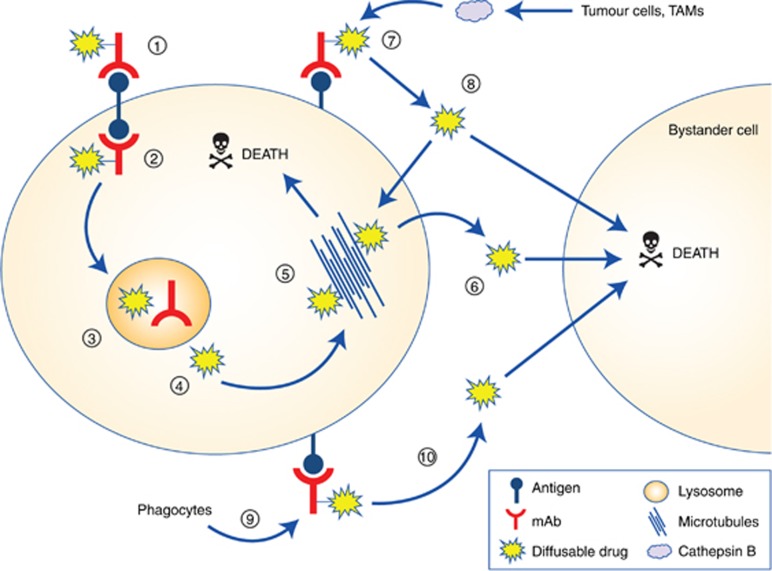
**Possible mechanism(s) of action are shown for an ADC with a diffusible drug (MMAE) attached with a cathepsin B cleavable linker.** The canonical processing pathway for an ADC involves (1) binding of the ADC to the target antigen and (2) internalisation. (3) The ADC is transported to the lysosome where the linker is cleaved, producing free diffusible drug (4). This free drug can then bind to microtubules (5) or DNA (depending on drug type) within the target cells to induce cell cycle arrest and ultimately result in cell death. The free drug can also diffuse out of the target cell (6) and penetrate surrounding ‘bystander’ cells to cause cell death. After ADC binding to the target antigen (7) but before internalisation, an alternative route for ADC processing is cleavage by extracellular enzymes (such as cathepsin B), which are released by surrounding tumour cells and tumour-associated macrophages (TAMs) and which generate diffusible drug from the ADC (8). This free drug can then penetrate surrounding ‘bystander’ cells resulting in cell death. Also, the ADC-bound target tumour cell may be internalised through Fc-mediated phagocytosis (9), which upon degradation of the target tumour cell would also result in release of free, diffusible drug (10).

The vc dipeptide linker, which is cleaved preferentially by the lysosomal protease, cathepsin B, is more stable in both human and mouse plasma than the pH-dependent hydrazone linker, resulting in lower toxicity in mice (Doronina *et al*, 2003). The vc linker has been commonly used to conjugate the tubulin-binding drugs MMAE and monomethyl auristatin F (MMAF) to mAbs. Although structurally similar, MMAE can readily enter cells via passive diffusion whereas MMAF contains a charged carboxylic acid terminus, which limits its passive diffusion into surrounding cells (Doronina *et al*, 2006). The canonical pathway involved in the intracellular processing of these particular linker/drug combinations first involves internalisation of the ADC, transportation to the lysosome where the linker is degraded by proteases including cathepsin B, resulting in the release of the highly potent, free drug. Then the drug binds to tubulins, and disrupts microtubule assembly to induce cell cycle arrest and ultimately cell death (Figure 2). Although the low cell permeability of MMAF limits its toxicity if the free drug is released from the ADC before the ADC reaches the target cell, MMAF-mediated killing is restricted to the target cell because MMAF cannot diffuse out of the cell, and thus cannot cause bystander killing. Consequently, MMAF ADCs require high tumour expression of target antigen to be effective (Smith *et al*, 2006), but are more potent than vc-MMAE ADCs when targeting internalising antigens *in vitro* (Sutherland *et al*, 2006). Conversely, as a free drug, MMAE is much more potent than MMAF (50–200-fold lower IC_50_) because of its increased cell permeability. Consequently, MMAE has the advantage of being able to diffuse out of the target cell and enter surrounding cells to cause bystander killing, something that MMAF cannot do.

These findings were reinforced by a detailed study of MMAE-containing ADCs, which showed that irrespective of the target antigen and its expression level, the loading ratio of drug on the antibody or the IC_50_ of the ADC, the concentration of MMAE released from the targeted cell best determined the extent of *in vitro* tumour cell killing (Li *et al*, 2016). Similarly, the intratumoural concentration of MMAE correlated with *in vivo* anti-tumour activity of the ADC. Given that these results were not reproduced with a MMAF-containing ADC, the membrane permeability of the released warhead and its ability to diffuse through the tumour seemed to be required for bystander killing.

Although internalisation of vc-MMAE-based ADCs provides one way in which they exert anti-tumour activity, studies indicate that ADC internalisation may not be required for anti-tumour action of these ADCs. For example, targeting the poorly internalised antigen CD21 with an ADC containing vc-MMAF was not effective, whereas conjugation of the same antibody with vc-MMAE showed potent anti-tumour activity in a preclinical lymphoma model (Polson *et al*, 2009), indicating that mechanisms independent of direct internalisation by antigen binding are at play. Furthermore, targeting of the non-internalising A1 domain of the glycoprotein Tenascin C with an intact antibody (or a SIP) using the vc linker and MMAE drug combination was effective, with the ADC format capable of producing cures in mice bearing human epidermal carcinoma and glioblastoma tumours (Gebleux *et al*, 2016). Substitution of a single amino acid of the dipetide linker of this ADC significantly altered the *in vivo* stability resulting in differences in activity of the ADC, and in some cases different cleaved drug products, whereas substitution with a non-cleavable linker abrogated any *in vivo* anti-cancer activity, confirming that a cleavable linker was essential for any anti-cancer activity of this non-internalising ADC (Dal Corso *et al*, 2017).

Although not antibody-based, small molecule drug conjugates that use an acetazolamide derivative to bind carbonic anhydrase at the tumour cell surface do not become internalised and are effective in treating renal carcinoma-bearing mice when linked via cathepsin-B cleavable dipeptide linkers to MMAE or the anthracycline analogue, PNU-159682 (Cazzamalli *et al*, 2016). In this case, the more serum-stable dipeptide linkers (valine–alanine and vc) were more active in reducing tumour growth compared with the less-stable valine–lysine or valine–arginine linkers when using the MMAE payload (Cazzamalli *et al*, 2017).

### Factors contributing to ADC-mediated bystander killing of non-internalising ADCs

As discussed above, ADCs employing a cathepsin B cleavable linker can be cleaved independently of direct antigen-internalisation or at least through alternative mechanisms. Cathepsin B expression is not limited to the lysosome, particularly in tumour cells. Tumour cells have increased membrane and secreted levels of cathepsin B, which is an attribute of their invasive phenotype (Poole *et al*, 1978; Buck *et al*, 1992; Sloane *et al*, 2005). Furthermore, tumour associated macrophages and stromal fibroblasts highly express cathepsin B (Campo *et al*, 1994; Reddy *et al*, 1995). The overproduction of cathepsin B by tumour cells and tumour-associated cells may at least be partly involved in cleavage of ADCs present in a tumour mass either in extracellular spaces or after target antigen binding (Figure 2). In support of this, conditioned media from tumour cell cultures contain cathepsins, which can cleave an ADC containing a dipeptide linker to release free drug and cause bystander killing (Lam *et al*, 2014). Furthermore, the cleavage of dipeptide linkers may not be exclusively performed by cathepsin B, as serine hydrolase carboxylesterase 1C can cleave the linker in rodent serum, resulting in ADC instability (Dokter *et al*, 2014; Dorywalska *et al*, 2016). Whether other enzymes common to the tumour microenvironment can also cleave dipeptide linkers extracellularly is not known.

The internalisation of ADCs after target antigen binding by tumour-associated myeloid or immune cells may also play a role in ADC catabolism. For example, a specific antibody bound to CD30^+^ tumour cells was internalised by macrophages, and macrophage-depletion reduced the anti-tumour efficacy of CD30-directed immunotherapy *in vivo* (Oflazoglu *et al*, 2007). Similar results have been seen with CD40-directed immunotherapy, with a combined depletion of NK, neutrophil and macrophages completely abolishing its anti-tumour activity (Oflazoglu *et al*, 2009). Furthermore, Li *et al*, 2017 have demonstrated that a non-binding, cleavable ADC was effective as *in vivo* treatment for human tumour xenografts, which had a significant content of tumour associated macrophages (TAMs). This anti-tumour effect was mediated by the Fc-mediated uptake and processing of the ADC by the TAMs, which then resulted in extracellular release of MMAE and bystander tumour cell killing. It is therefore clear that immune cells, in particular macrophages, are required for immunotherapeutic efficacy, and these effects may be dependent on Fc-mediated phagocytosis. However, ADCs with site-directed mutation to reduce Fc-binding still show efficacy, meaning that in some cases Fc-dependent effector functions are not solely required for tumour responses to ADCs (McDonagh *et al*, 2008).

## Conclusion

ADCs are revolutionising the field of cancer treatment by harnessing the specificity of tumour-targeting mAbs with the stability and high potency of advanced linker/drug combinations. Although it has been established that internalisation and intracellular processing of ADCs provide one mechanism of action, it is becoming clear that this is not the only mechanism of ADC action and other processing mechanisms independent of direct cell-surface antigen internalisation can produce effective ADCs.

Depending on the linker type and drug combination, non-internalising ADCs may have additional advantages by exploiting pathological features inherent to the microenvironment of many tumours such as hypoxia, necrosis, excess reducing equivalents, acidity, an abundance of both active extracellular proteases and protease-rich tumour-infiltrating myeloid cells. By providing the setting for conditional activation of ADCs, tumour properties other than antigen specificity can enhance the tumour selectivity of ADCs. Finally, a strong rationale exists for combining ADCs with other cancer therapies that either act upon or react to elements of the tumour microenvironment. For example, cytotoxic chemotherapy drugs can increase tumour expression of ADC-catabolising cathepsin B (Shree *et al*, 2011). In addition, tubulin-targeting or DNA-binding warhead drugs consequently released in the tumour microenvironment can be immunostimulatory and thus used to engage the therapeutically beneficial effects of immune checkpoint inhibitors (Muller *et al*, 2014; Gerber *et al*, 2016; Rios-Doria *et al*, 2017).

## Figures and Tables

**Figure 1:**
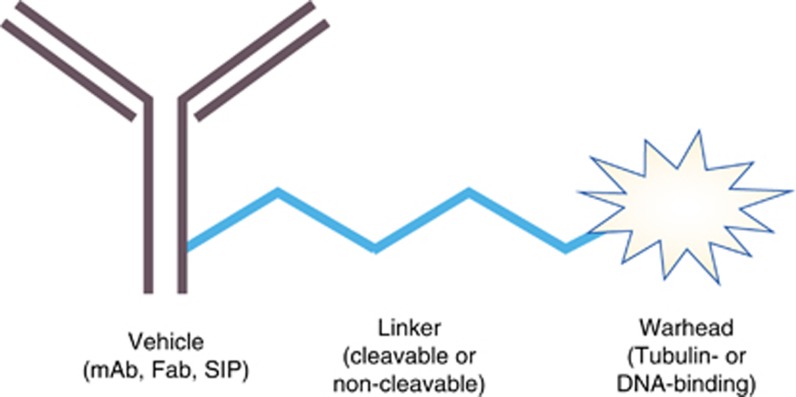
**Schematic diagram of antibody drug conjugate.** An antibody drug conjugate consists of a cancer-targeting vehicle, usually the whole or fragment of a monoclonal antibody, connected by a cleavable or non-cleavable linker to a potent cytotoxic warhead drug. mAb, monoclonal antibody; Fab, fragment antigen binding; SIP, small immune protein.
